# Are Horse Owners Able to Estimate Their Animals’ Body Condition Score and Cresty Neck Score?

**DOI:** 10.3390/vetsci9100544

**Published:** 2022-10-03

**Authors:** Sara Busechian, Luca Turini, Micaela Sgorbini, Camillo Pieramati, Lorenzo Pisello, Simona Orvieto, Fabrizio Rueca

**Affiliations:** 1 Department of Veterinary Medicine, University of Perugia, Via San Costanzo 4, 06126 Perugia, Italy; 2 Department of Agricultural, Food and Agro-Environmental Sciences, Via del Borghetto 80, 56124 Pisa, Italy; 3 Department of Veterinary Sciences, University of Pisa, Viale delle Piagge 2, 56124 Pisa, Italy; 4 Independent Researcher, 06100 Perugia, Italy

**Keywords:** horse, body condition score, cresty neck score, overconditioning

## Abstract

**Simple Summary:**

Obesity and overconditioning are becoming more prevalent in horses and could be the cause behind various diseases. Evaluation of body condition scores (BCS) and cresty neck scores (CNS) are important tools used to assess the risk of obesity, and owners and caretakers should be able to correctly evaluate their animals. The aim of this study was to compare the ability of owners to correctly assess the BCS and CNS of their animals, comparing them with the scores given by an experienced examiner. In our population, overconditioning was present in 29% and 24% of horses according to the owners and the vet, respectively, while obesity was detected by the owners and the vet in 2% and 1% of cases, respectively. Agreement between the owners and the vet was slight for both body condition score and cresty neck score, and it was influenced by age, breed, gender, and activity of the horse. Education of owners and caretakers to correctly assess their animals is important to prevent the development of obesity and overconditioning and related diseases.

**Abstract:**

Obesity and overconditioning are becoming more prevalent in horses, and are influencing the animal’s health, activity, and reproductive efficiency. Body condition score (BCS) and cresty neck score (CNS) have been correlated with the development of various diseases. Many of the papers in the literature evaluate the prevalence of obesity and overconditioning in horse populations considering BCS given by owners. The aim of this study was to evaluate the validity of the owners’ estimation of the degree of equine obesity or overconditioning by comparing BCS and CNS provided by each owner and a board-certified vet to a cohort of 259 horses and their agreement based on age, breed, sex, and activity of the animals. Overconditioning was present in 29% and 24% of horses according to the owners and the vet, respectively, and obesity in 2% and 1%, respectively. Agreement between BCS and CNS given by the owners and the vet was slight, with owners scoring horses either lower or higher than the vet. Agreement between the owners and the vet assessment was also influenced by age, breed, sex, and activity of the animals. Education of owners and caretakers to correctly assess BCS and CNS and regular evaluation of the animals throughout the year is important to prevent the development of obesity and overconditioning and related diseases.

## 1. Introduction

Horses’ health, productivity, and reproductive efficiency all depend on their body condition, which is described as the ratio of body fat to non-fat components in their bodies [1,2,3,4]. Body fat storage is significant in all healthy animals because it symbolizes the amount of energy reserves the animal has during periods of low energy availability. In the United States and Europe, overweight and obese horses are becoming more common. According to the literature, generally in various populations and areas of the world, more than 18% of horses are overweight/obese; this could be due to an imbalance between energy intake and expense [5,6,7,8,9,10]. Excessive bodyweight (BW) can prompt medical problems including insulin resistance and equine metabolic syndrome, both of which can cause poor thermoregulation, laminitis, and impaired athletic performances in horses [7,10,11,12,13,14,15]. To avoid this, numerous approaches for evaluating the horse’s stored energy reserves, both subjectively and objectively, have been developed. The body condition score (BCS) and the cresty neck score (CNS) are two of the most utilized approaches in the literature [3,4,16,17,18,19]. Body condition score reflects the degree of apparent adiposity of specific anatomical sections of the horse, and it is assessed visually and tactilely on a scale ranging from 1 to 9 [17] or from 1 to 5 [19]. Low levels indicate emaciation, whereas high values indicate obesity, regardless of the scale employed. Veterinary practitioners believe the BCS to be a valuable tool for detecting obesity and determining the risk of laminitis in horses [20]. The cresty neck score is a descriptive, ordinal scale used to determine regional adiposity by measuring nuchal fat accumulation (around the top of the neck) [18]. The quantity of nuchal fat is disproportionate to the amount of total body fat, and it can, therefore, be used as a separate indicator of adiposity [21]. A recent paper indicated that CNS is a better indicator of insulin dysregulation in ponies compared to BCS [16]. Bodyweight estimation is crucial for managing a horse’s health and nutrition; however, few horse owners have access to a scale or can precisely estimate the BW of their horses. A few papers [12,14,22,23,24] have considered the prevalence of obesity and overconditioning in horses in various regions of the world, basing their assessment on questionnaires administered to owners. Owners, though, can underestimate the BCS of their horses [12,23], and these papers should be considered based on these findings. The aim of our study was to determine the prevalence of overconditioning/obesity in a population of leisure and retired horses, and to see if the owners were able to estimate the BCS and CNS of their animals, comparing it to the expert observer (VET) scores. Finally, the study tried to evaluate the agreement between the owners’ and the vet’s estimations based on the horses’ breed, age, gender, and activity.

## 2. Materials and Methods

### 2.1. Horses and Management

A cohort of 259 adult horses belonging to 30 different owners were enrolled in the study. The oral consent of the owners was obtained. The study was carried out according to Directive 2010/63/EU. The research was conducted under farm conditions; thus, no experimental animals were included and, therefore, approval from an Ethical Committee was not required. Animals were not treated with any kind of medication, and evaluation of BCS and CNS is commonly used in equine practice.

Although all the animals were given little or no activity at the time of the evaluation, the stabling conditions and management were different. The majority were housed in stables, with paddock turnout once or twice a day, for at least a couple of hours each time. The feeding ration was comprised of first-cut grass hay, mostly two or three times a day, and a small amount of sweet feed (mixes of grains, corn, bran, molasses, minerals, and vitamins). The feeding ration might have slightly varied according to the animal’s requirement. Racehorses were not in active training at the time of the interview.

**Figure 1:**
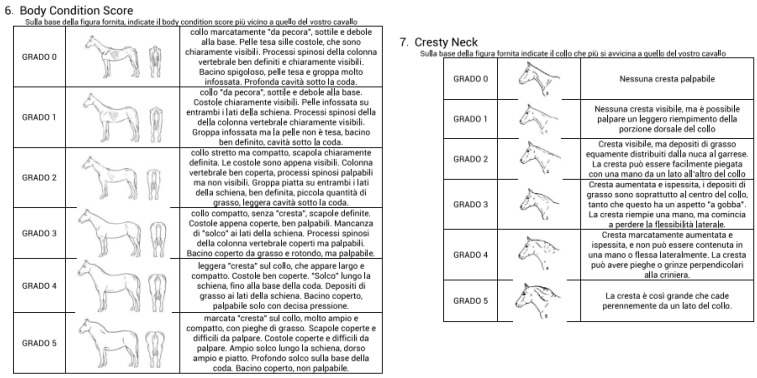
Diagram presented to the owners and the vet to evaluate BCS and CNS of the horses in the study. Modified from Refs. [18,19].

Body Condition Score (BCS) was determined on a scale ranging from 0 to 5 according to Carroll and Huntington [19] and Cresty Neck Score (CNS) on a scale from 0 to 5, according to Carter et al. [18]. The scores were always evaluated by the same vet (SB) and by each owner, in a blinded manner between each other. The owners were not educated before the survey, and none of them had any background in veterinary medicine or related disciplines that could have prepared them for evaluating BCS and CNS. All owners considered themselves to have extensive knowledge in equitation and horsemanship, having owned or ridden horses for at least 10 years. A picture representing the scale for BCS and CNS with a small written description in Italian was given to the owners and the vet during the interview, asking them to place an X on a visual analogue scale (Figure 1). No observer was allowed to touch the horse during the examination, but they could walk around the animal to better evaluate their morphology.

At the same time, a survey was administered to each owner to determine the age, sex, breed, and type of activity of the horse. Activity was considered as what the horses were trained for, even if not in active training at the time of the interview. Animals were divided into three categories based on their age: young (0–10 years old), adult (11–20 years old), and geriatric (21–30 years old). Female, male, and gelding were used to define the animals’ sex. Breeds were grouped as Fullblood (FB) (e.g., Arabians, Thoroughbreds, Standardbreds, Quarter Horses), Warmblood (WB) (e.g., saddlebreds), Baroque, Coldblood (e.g., draught breeds, Haflingers), and Ponies [25]. To categorize the animals in the activity groups “Breeding”, “driving”, “lessons”, “trekking”, “endurance”, “racing”, “western”, “start training”, “jumping”, “show”, and “nothing” were used.

### 2.2. Statistical Analysis

The prevalence for BCS and CNS according to the owner’s and the vet’s estimation and for age, sex, breed, and type of activity were calculated.

To verify the influence of the operator’s experience in the evaluation of the BCS and CNS, all the animals were evaluated by the owners and compared with the assessment performed by an expert observer (vet) (SB).

The agreement between the owners and the vet was estimated by Cohen’s kappa coefficient. The calculation of the coefficient was performed on the whole dataset and on different subsets based on age, breed, sex, and type of activity of the horse.

The Cohen’s kappa coefficient result was interpreted as follows: values ≤ 0 indicated no agreement, 0.01–0.40 slight, 0.41–0.60 moderate, 0.61–0.80 substantial, 0.81–1.00 optimal, NE: not evaluable. A pairwise spearman rank correlation was used to evaluate the correlation between the estimations made by the owners and the vet. Statistical analysis was performed using JMP software (SAS Institute Inc., Cary, NC, USA).

## 3. Results

According to age, the cohort of horses included were composed by 163/259 (62.9%) young, 77/259 (29.7%) adult, and 19/259 (7.4%) geriatric animals. Of the 259 horses included, 25/259 (9.6%) were males, 153/259 (59.1%) female, and 81/259 (31.3%) geldings. According to breed, 126/259 (48.6%) were Fullblood, 92/259 (35.5%) Warmblood, 18/259 (7.0%) Ponies, 13/259 (5.0%) Coldblood, and 10/259 (3.9%) Baroque. In Table 1, the number and prevalence of horses grouped according to the type of activity are reported.

**Figure 2:**
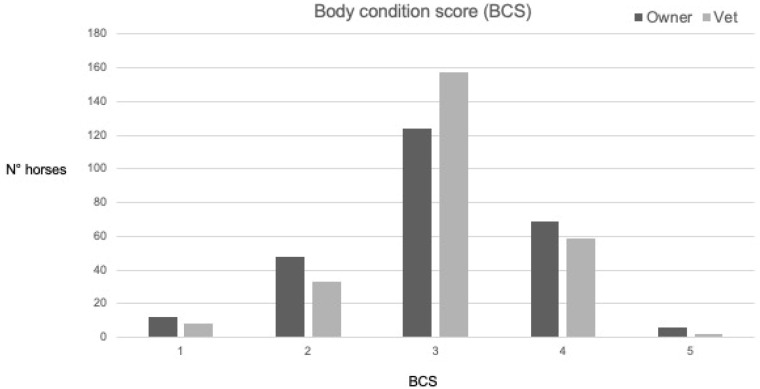
Data regarding the distribution of BCS in a scale from 1 to 5 performed by the owners or by the expert observer (vet) for all 259 horses included in this study. Legend: BCS—Body Condition Score [19].

**Figure 3:**
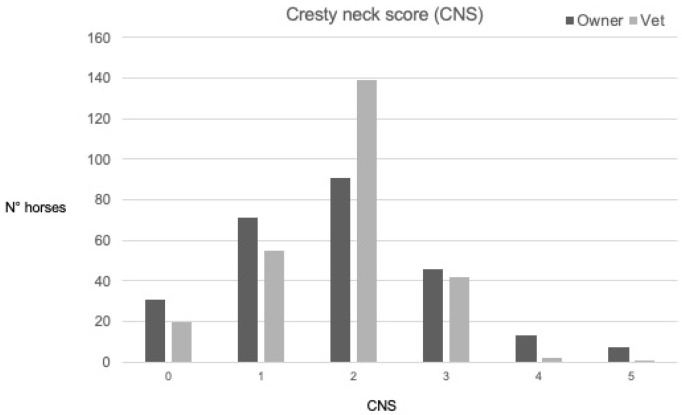
Data regarding the distribution of CNS in a scale from 0 to 5 performed by the owners or by the expert observer (vet) for all 259 horses included in this study. Legend: CSN—Cresty Neck Score [18].

Data regarding the distribution of BCS and CNS made by the owners and the vet are reported in Figure 2 and Figure 3, respectively. Considering the owners’ responses, 69/259 (29%) horses were considered overconditioned, (BCS at least 4/5), and 6/259 (2%) obese (BCS 5/5), while according to the vet, 59/259 (24%) horses were overconditioned and 2/259 (1%) obese. The agreement between the owners and the vet was considered slight for the cohort of horses evaluated for BCS and CNS and for most of the evaluation based on age, sex, breed, and type of activity.

Results for Cohen’s kappa coefficient calculated for the cohort of horses (259 horses) and for the horses classified for age, gender, breed, and type of activity are reported in Table 2.

The correlation between CNS and BCS made by the vet was 0.48 (*p*-value < 0.001).

## 4. Discussion

BCS and CNS are considered good indicators of the level of obesity and overconditioning of horses [11,16,18,26]. These last two situations could predispose horses to severe health problems such as equine metabolic syndrome, insulin dysregulation, hyperlipemia, and laminitis [11,16,27]. To help prevent these diseases, owners and caretakers should be able to critically appraise the BCS and CNS of their animals. Considering the grades given by the vet, the correlation between BCS and CNS is moderate, making it important to use both grading systems when evaluating a horse: according to the literature, CNS is a better indicator of insulin dysregulation, and its determination is important to monitor the risk of Equine Metabolic Syndrome [11,16].

The prevalence of overconditioning and obesity in our population (29% and 2% according to the owners and 24% and 1% according to the vet) is similar to what can be found in the literature [10], but a comparison is made difficult by the different scoring systems used and the diverse definition of overconditioning and obesity used in the papers. Obesity, though, seems to be less present than in other equine populations, but this could be related to the different breeds included in our study: the low number of ponies (18/259, 7%), considered more at risk for obesity and overconditioning than other breeds [10,13,28], can influence the number of obese animals in our population. In other studies, ponies represented a higher proportion of the number of animals evaluated [12,13,14], but in our cohort, the BCS of ponies was distributed among all grades, and not limited to the higher ones. At the same time, other breeds commonly considered overweight, such as Quarter Horses and Arabians, were used for light work, making them less likely to increase their body weight [10,28].

Overall, the agreement between observers was considered slight; owners tended to score horses either lower or higher than the vet, and they found a lower number of horses scored 3/5, compared to the vets, as owners tended to give scores either higher or lower than those assigned by the vet (see Figure 2 and Figure 3). Our study showed that the agreement between the observation done by the owners or by the vet could be influenced by the age, sex, breed, and type of activity. The Cohen’s kappa coefficient tended to increase with the age of the horses, and it changed from a slight agreement to a moderate agreement regarding the CNS. In our study, the geriatric horses had a lower BCS compared to the young and adult horses. Perhaps this difference could explain the better agreement between the owners and the vet. Geriatric horses have lived with their owners longer than the others, and their caretakers were most likely more used to evaluating their BCS and CNS to monitor the wellbeing of their animals, especially when affected by chronic diseases.

The horse’s breed seems to influence the agreement between owner and vet. Particularly, the owners of WB horses had a moderate agreement for BCS, while owners of Baroque and FB horses showed a moderate agreement for BCS and a moderate agreement for CNS. This could be due to the type of breeds included in the Baroque and FB groups. In particular, Arabians and Spanish horses showed higher CNS because of their morphology [29,30] and owners that buy this type of horse, especially for showing and breeding, tend to be more aware of this inherent trait. Furthermore, the Baroque and FB breeds can be used for show, and they are therefore usually bred or bought for their specific morphologic characteristics, which are assessed by focusing the attention on the body areas usually evaluated for BCS. It is therefore possible that owners of these breeds have a more trained eye, which allows them to better evaluate the BCS and CNS of their horses.

The type of activity influenced the degree of agreement between the owners and the vet in the evaluation of BCS and CNS. In fact, owners of western horses had an optimal degree of agreement with the vet for BCS, while others had no or slight agreement between the owners and the vet. This could be related to the owners being more trained on evaluating morphological characteristics. The management of pleasure horses emphasizes the attitude of keeping horses in a “good” condition (BCS 4/5) [13]. The literature reported that Quarter Horses showed the highest prevalence of being overweight [12], thus Quarter Horse owners may be more accustomed to face the morphologic evaluation of their animals, also for nutrition management purposes.

Owners were not educated by the interviewer before being presented with the scoring systems and had no background on veterinary medicine and related disciplines, because the aim of the study was to evaluate their ability to identify correctly the BCS and CNS of their horses. All the owners that were interviewed considered themselves experts in equitation and horsemanship, having owned or ridden horses for at least 10 years. The slight agreement between an experienced observer and the owner should be kept in mind, especially when considering the prevalence of obesity from the owner’s perspective. The results are consistent with other studies [12,23] and highlight the difficulties that owners could have in correctly estimating the risk of their horses developing obesity-related diseases, such as laminitis, and the importance of educating owners and caregivers to correctly estimate the BCS and CNS of their horses. This, coupled with regular evaluation of the horses, could prevent the development of obesity and overconditioning and related diseases.

Only one vet was evaluating all horses: this is similar to what usually happens in equine clinical practice, when one vet is responsible for the care of multiple horses in different premises. It is therefore important that owners and caretakers are able to evaluate the welfare of each horse, to prevent or identify possible diseases early on. A possible bias related to the different experience of the owners was reduced, including only people with at least 10 years of experience in horsemanship.

## 5. Conclusions

In our cohort of non-athlete horses, the prevalence of overconditioning and obesity based on owners’ perception was 29% and 2%, and, based on the vet’s assessment, was 24% and 1%. The agreement between the owners and the vet when assessing BCS and CNS was slight, and was also influenced by the age, breed, sex, and activity of the horse. Owners should be educated in the correct evaluation of their horses and are advised to perform regular determinations of both BCS and CNS, especially in breeds that are predisposed to EMS and laminitis.

## Figures and Tables

**Table 1:** Number (N) and prevalence (%) of horses included according to the type of activity.

Activity	N/259 (%)
Breeding	63 (24.3%)
Driving	10 (3.9%)
Endurance	7 (2.7%)
Jumping	33 (12.7%)
Leisure	30 (11.5%)
Lessons	58 (22.4%)
Racing	34 (13.1%)
Show	3 (1.2%)
Start training	10 (3.9%)
Trekking	8 (3.1%)
Western	3 (1.2%)

**Table 2:** Cohen’s kappa (K) coefficient (% of discordant cases), degree of agreement (A), and Spearman rank correlation (ρ) calculated for BCS and CNS on the cohort of horses included and according to age, sex, breed, and type of activity. Legend: BCS: Body Condition Score; CSN: Cresty Neck Score.; No: values ≤ 0 indicated no agreement; Slight: values between 0.01 and 0.40 indicate slight agreement; Moderate: values between 0.41 and 0.60 indicate moderate agreement; Optimal: values between 0.81 and 1.00 indicate optimal agreement; NE: not evaluable. *: *p*-value between 0.05 and 0.01; **: *p*-value between 0.01 and 0.001; ***: *p*-value < 0.001; NS: not significant.

	BCS			CNS		
	K	A	ρ	K	A	ρ
Cohort (n = 259)	0.32 (42.5%)	Slight	0.53 ***	0.35 (46.7%)	Slight	0.66 ***
**Age**						
Young (n = 163)	0.25 (39.9%)	Slight	0.36 ***	0.31 (48.4%)	Slight	0.60 ***
Adult (n = 77)	0.38 (42.9%)	Slight	0.70 ***	0.39 (44.1%)	Slight	0.73 ***
Geriatric (n = 19)	0.41 (42.1%)	Moderate	0.82 ***	0.40 (42.1%)	Slight	0.80 ***
**Sex**						
Female (n = 152)	0.33 (40.8%)	Slight	0.53 ***	0.40 (45.4%)	Slight	0.72 ***
Gelding (n = 81)	0.31 (45.7%)	Slight	0.57 ***	0.21 (49.4%)	Slight	0.51 ***
Male (n = 25)	0.27 (44.0%)	Slight	0.42 *	0.29 (48.0%)	Slight	0.60 **
**Breed**						
Baroque (n = 10)	0.15 (0.50%)	Slight	0.23 ^NS^	0.42 (40.0%)	Moderate	0.45 ^NS^
Coldblood (n = 13)	0.11 (53.8%)	Slight	0.54 ^NS^	−0.10 (69.2%)	No	−0.01 ^NS^
FB (n = 126)	0.23 (44.4%)	Slight	0.45 ***	0.45 (38.9%)	Moderate	0.67 ***
Pony (n = 18)	0.14 (66.7%)	Slight	0.65 **	0.01 (77.8%)	Slight	0.65 **
WB (n = 92)	0.45 (32.6%)	Moderate	0.65 ***	0.24 (48.9%)	Slight	0.54 ***
**Activity**						
Breeding (n = 63)	0.21 (42.8%)	Slight	0.31 *	0.56 (33.3%)	Moderate	0.80 ***
Driving (n = 10)	−0.22 (0.60%)	No	0.00 ^NS^	0.10 (60.0%)	Slight	0.46 ^NS^
Endurance (n = 7)	−0.04 (57.1%)	No	0.24 ^NS^	0.16 (42.8%)	Slight	0.35 ^NS^
Jumping (n = 33)	0.45 (27.2%)	Moderate	0.59 ***	0.21 (48.5%)	Slight	0.51**
Leisure (n = 30)	0.39 (43.3%)	Slight	0.68 ***	0.30 (50.0%)	Slight	0.74 ***
Lessons (n = 58)	0.35 (46.5%)	Slight	0.69 ***	0.23 (55.2%)	Slight	0.61 ***
Racing (n = 34)	0.05 (41.1%)	Slight	0.18 ^NS^	0.26 (44.1%)	Slight	0.43 *
Show (n = 3)	NE (66.7%)	NE	0.50 ^NS^	NE(100%)	NE	0.50 ^NS^
Start training (n = 10)	0.17 (0.50%)	Slight	−0.09 ^NS^	0.33 (40.0%)	Slight	0.61 ^NS^
Trekking (n = 8)	0.43 (37.5%)	Moderate	0.81 *	-0.25 (62.5%)	No	0.14 ^NS^
Western (n = 3)	1(0.0%)	Optimal	1.00 ***	0.40 (33.3%)	Slight	0.50 ^NS^

## Data Availability

The data are available by sending an email to the corresponding author.
